# Microsurgical techniques in periodontics - A review

**DOI:** 10.6026/973206300222690

**Published:** 2026-04-30

**Authors:** Anand Bhalodi, Priyangha Ravichandran, Praveena Paliwal, Vidyalakshmi Shankararaman, Sangita Bhalodi, Eshita Patel, Utsav Patel

**Affiliations:** 1 Department of Periodontology, Pacific Dental College and Hospital, Udaipur, Rajasthan, India; 2 Department of Orthodontics, Chettinad Dental College, Tamil Nadu, India; 3 Department of Dentistry, Eternal Dental, 530 W. Eaton Ave, Suite L, Tracy, CA 95391, United States; 4 Department of Periodontology, Thai Moogambigai Dental College and Hospital, Mugappair, Chennai, Tamil Nadu, India; 5 Department of Periodontology, Karnavati School of Dentistry, A/907, Uvarsad, Gandhinagar, Gujarat, India; 6 Department of Periodontology, AMC Dental College and Hospital, Khokhra, Ahmedabad, Gujarat, India; 7 Department of Periodontology, Pacific Dental College, Udaipur, Rajasthan, India

**Keywords:** Microsurgery, loupes, periodontics, tissue management

## Abstract

Since the earliest days of dentistry, professionals have recognized the importance of magnification in improving clinical precision. Advances in intraoral magnification, including loupes and operating microscopes, have ushered in the era of micro-dentistry and transformed dental practice. The effective use of these technologies requires a high level of skill and expertise to achieve optimal treatment outcomes in periodontics. However, microsurgical procedures such as connective tissue grafts and periodontal flap surgeries remain technically challenging due to increased instrument complexity and a restricted field of vision, along with limited precise documentation of techniques. Thus, enhanced tissue management practices support the development of the emerging subspecialty of microsurgical periodontics.

## Background:

Utilizing the surgical microscope, which enables enhanced visual clarity, illumination and precision during operative procedures, periodontal microsurgery represents a significant advancement over conventional surgical techniques. The integration of magnification and coaxial illumination has fundamentally transformed the manner in which delicate periodontal tissues are handled, allowing clinicians to perform procedures with greater accuracy and minimal trauma. These developments would not have been possible without the incorporation of the surgical microscope into periodontal practice [1, 2]. Over the past several decades, the specialty of periodontics has experienced remarkable progress, particularly in surgical interventions demanding refined technical skill and superior visual discrimination [3]. Procedures such as guided tissue regeneration, esthetic crown lengthening, gingival augmentation, soft and hard tissue ridge augmentation, osseous resection and implant placement necessitate meticulous tissue manipulation and precise suturing techniques. These interventions often involve fine anatomical structures and limited surgical fields, where even minimal inaccuracies may compromise esthetic and functional outcomes. Therefore, enhanced magnification becomes indispensable to achieve optimal clinical results. From a biological perspective, magnification supports minimally invasive principles by facilitating atraumatic handling of tissues, precise incision placement and accurate wound approximation. When tissues are managed with minimal trauma and exact primary closure is achieved, healing predominantly occurs by primary intention rather than through secondary healing characterized by granulation tissue formation.

Consequently, magnification plays a pivotal role in reducing post-operative inflammation, minimizing scarring and accelerating tissue recovery [3]. Nevertheless, an important optical consideration in magnification systems is the inverse relationship between magnification and field of view. As magnification increases, the visible operative field correspondingly decreases. In periodontal practice, clinicians frequently need to visualize adjacent teeth, surrounding gingival architecture and overall occlusal relationships. Excessive magnification may limit spatial orientation and compromise ergonomic efficiency. Hence, extremely high magnification levels are not always practical for routine periodontal surgeries [4, 5- 6]. Conversely, in highly focused esthetic procedures involving a single tooth or a confined surgical site such as papilla reconstruction or microsurgical root coverage-greater magnification can be advantageously employed. In such scenarios, the restricted operative field is acceptable because the clinician's attention is concentrated on a limited anatomical area requiring extreme precision. For many periodontal surgical procedures, prism telescopic loupes with approximately 4x magnification provide an optimal balance between magnification, field of view and depth of field. This level of magnification enhances visual acuity sufficiently to improve surgical precision while maintaining adequate spatial awareness and ergonomic comfort. Therefore, it is of interest to review current developments in microsurgical techniques in periodontics.

## Mucogingival procedures:

When it came to covering roots, lateral flaps, free gingival grafts and coronal advanced flaps were all utilized; nevertheless, the outcomes of these procedures were not uniform. A comprehensive analysis of research on root coverage revealed that the connective tissue graft technique, which was initially presented by Raetzke in 1985, is the procedure that is both the most effective and the most reliable [7]. When compared to macroscopic connective tissue grafts, microsurgical and macro-surgical grafts shown to have superior vascularization and root coverage qualities [7, 8].

## Microsurgery for the grafting of connective tissue:

A wide variety of macro-surgical procedures for connective tissue transplant locations have been described by the authors. A "box" or flap, as well as flaps that were sulcular and laterally positioned, were utilized in order to cover the connective tissue graft. Since 1989, Langer and Langer have been responsible for the development of a revolutionary connective tissue graft method [9]. As highlighted by Burdhardt and Land (2005), the enhancements that were made to this traditional procedure through the application of micro- surgical techniques made it possible to improve the placement and suturing of the incisions, which ultimately led to more positive outcomes [10]. A method that is sulcular, also known as flapless, can be helpful in minimizing recession. Sulcular incisions made with a crescent knife facilitate the separation of tissue following treatment of the root with citric acid and/or tetracycline. The pouch that is produced using this procedure is suitable for the transplant. Before the graft is placed inside the pouch, it is meticulously measured to ensure that it is three millimeters wider and three millimeters longer than the recession defect. A close bond between the graft and the root surface is created by the micro-surgical suturing sling, which helps to facilitate integration. For this particular operation, a 7-0 or 8-0 suture needle and a spatula needle are seen in the image [11, 12].

## Microsurgical techniques for periodontal flaps:

In the past, one of the most common errors that occurred in the construction of flaps was the practice of making incisions that were too small. This can make it difficult to gain access and impede treatment. When it comes to flap margins and closures, the utilization of loupes can greatly improve the precision of the area. This can be accomplished through the technique of careful dissection of a periodontal flap that has a scalloped butt-joint margin and a uniform thickness. Consequently, this ensures that the tissue is aligned correctly with the teeth or the opposing flap in a location that is toothless. Incisions made with carbon steel blades are precise and crisp and they are made at right angles to the surface tissue. A sterile razor blade of surgical quality is broken down into this blade, which is then broken down to the required size. In order to ensure that the thickness of the flap remains maintained throughout the dissection process, additional incisions are made at right angles to the primary cut. During this technique, a double-edged ophthalmic blade is utilized to ensure excellent precision. Through the utilization of loupes and a magnification of four times, it is able to efficiently manage exposure while simultaneously decreasing the amount of harm that is inflicted onto the tissue. In order to accomplish this, it is necessary to avoid stretching, distorting, or ripping the flap. This is especially important when using miniature microsurgical instruments that have been suitably constructed [13]. The extraordinary sharpness of these blades, in addition to their tiny dimensions is the distinguishing characteristics of these knives. Because of this, it is possible to make precise cuts and motions in restricted spaces.

## Microsurgical techniques for root preparation:

In dental offices, stereomicroscopy is routinely used to determine whether or not residual calculus is present after scaling and surgical procedures have been performed. According to the findings of a number of researchers, the rigorous debridement of the root surface is the most important step in attaining successful periodontal therapy. Those who were subjected to surgical access exhibited significantly lower levels of residual calculus, ranging from 14% to 24%, in comparison to their counterparts who were treated without surgical intervention, who showed levels ranging from 17% to 69%. This was determined by comparing the root calculus residuals after scaling and root preparation [14, 15]. Despite the fact that there are not many studies that compare root preparation during surgical access with and without magnification, the use of a surgical operating microscope has the potential to significantly increase a surgeon's ability to perform this essential procedure.

## Conclusion:

It is necessary for periodontal surgeons to have a high level of expertise in periodontal microsurgery in order to preserve their recognized status as specialists in the meticulous treatment of both soft and hard tissues. Those persons who devote themselves to studying the principles and procedures of microsurgery are able to access a new dimension because to the greater visual clarity that magnification provides. When these ideas are incorporated into the techniques that are already used for periodontal surgery, it represents a progression toward treatments that are less invasive and traumatic, which in turn promotes faster recovery times. This exceptional acceptance of microsurgery among patients has occurred in the context of today's scenario, which is characterized by increased patient knowledge.
